# Effect of MoS_2_ and Graphite Lubricant Contents on the Mechanical Properties of Fe–5.0 wt.%Si Soft Magnetic Composites

**DOI:** 10.3390/ma19122649

**Published:** 2026-06-19

**Authors:** Jehyeon Park, Seonbong Lee

**Affiliations:** 1 Department of Mechanical Engineering, Keimyung University, Daegu 42601, Republic of Korea; 5648967@stu.kmu.ac.kr; 2 Department of Automotive Engineering, Keimyung University, Daegu 42601, Republic of Korea

**Keywords:** Fe–5.0 wt.%Si SMC, MoS_2_/graphite lubricant, high-temperature compaction, finite element analysis, densification uniformity, Vickers hardness, one-way ANOVA, hydrostatic stress, toroidal core

## Abstract

This study investigated the effect of MoS_2_/graphite lubricant composition on the high-temperature compaction behavior, local mechanical uniformity, and microstructural characteristics of Fe–5.0 wt.%Si SMCs. Nine lubricant compositions were prepared by varying MoS_2_ and graphite contents, and their friction behavior, Vickers hardness, and compaction behavior were evaluated experimentally and by FEA. One-way ANOVA confirmed that lubricant composition significantly affected the Vickers hardness response (F = 4.245, *p* = 0.000273). The measured friction coefficients were applied as interface friction conditions in FEA, and the relative density, effective strain, and absolute hydrostatic stress distributions were compared. Among the investigated compositions, C3, containing 1.0 wt.% MoS_2_ and 0.3 wt.% graphite, showed the lowest friction coefficient and Vickers hardness standard deviation. In FEA, C3 also showed balanced relative density, effective strain, and hydrostatic stress distributions. XRD confirmed the α-Fe-based bcc Fe–Si matrix, while SEM-EDS indicated locally distributed lubricant-derived residual regions. Therefore, C3 was selected as the most balanced lubricant composition within the investigated range. Future studies will evaluate electromagnetic properties, including core loss and magnetic permeability.

## 1. Introduction

Axial flux permanent magnet (AFPM) motors for electric vehicles have attracted attention as next-generation drive systems because they can achieve high torque density and short axial length [1,2]. The stator core of an AFPM motor forms axial magnetic flux and three-dimensional magnetic flux paths; therefore, conventional laminated electrical steel has limitations in terms of shape flexibility and magnetic flux path design [1,3]. Soft magnetic composites (SMCs) are powder metallurgy-based materials fabricated by compacting insulated metal magnetic powders and are suitable for producing complex three-dimensional core geometries [3,4]. Owing to these characteristics, SMC cores have been evaluated as candidate materials for stator cores in AFPM motors for electric vehicles [3,5].

Fe–Si-based SMCs are known to exhibit high magnetic flux density and soft magnetic properties; however, powder brittleness and forming resistance increase with increasing Si content [6,7,8]. During high-density compaction, increased powder–die and powder–powder friction can lead to nonuniform powder rearrangement and densification, which may result in density variation and scatter in local mechanical properties within the compact [9,10,11]. In SMC compaction, the lubricant is a key variable that directly affects powder flowability, die-wall friction, particle rearrangement, and final densification behavior [8,12]. An appropriate amount of lubricant reduces forming resistance and promotes densification, whereas excessive addition can hinder contact between metal particles and cause pore formation or reduced densification [8,12,13]. For stable high-temperature compaction of Fe–Si-based SMCs, lubricant composition design suitable for the target powder and forming conditions is required [6,8,12,13].

Recent lubricant additive studies have reported that micro-scale solid additives can reduce friction and improve contact stability under severe contact conditions [14,15,16,17]. These studies provide useful background for understanding the role of solid lubricant additives. The present study focuses on micro-scale MoS_2_/graphite solid lubricant composition design for high-temperature compaction of Fe–5.0 wt.%Si SMCs.

In powder metallurgy, conventional lubricants such as zinc stearate have been widely used to improve powder flowability, compactability, and ejection behavior [18]. However, such organic lubricants are generally associated with green compaction and sintering-based powder metallurgy routes, and their direct application to high-temperature compaction requires caution because thermal removal or degradation can occur during heating [16,18]. Therefore, the MoS_2_/graphite system was selected in this study not as the only possible lubricant system, but as a representative micro-scale layered solid-lubricant combination suitable for evaluating lubricant composition effects under the high-temperature compaction condition of Fe–5.0 wt.%Si SMCs [14,16,17,19,20]. This selection also allows the complementary roles of a sulfide-based layered lubricant and a carbon-based layered lubricant to be evaluated while maintaining fixed insulation and binder conditions [14,19,20].

MoS_2_ and graphite are representative layered solid lubricants that contribute to friction reduction through low interfacial shear resistance [14,15,16,17,20]. MoS_2_ exhibits reduced shear resistance due to its layered structure; however, when it reacts with an Fe-based matrix at high temperature, it can be converted into FeS or Fe–Mo–S-based sulfides, resulting in consumption of the original lubricating phase [19,20]. Graphite has high chemical stability and thermal conductivity and can complement the lubricating role of MoS_2_ at high temperature [17,20]. Previous Fe–MoS_2_-based composite studies reported that graphite addition can delay the reaction between MoS_2_ and the Fe matrix and improve friction reduction behavior [19,20]. This reaction-delay mechanism is used in the present study as a literature-based background for designing the MoS_2_/graphite lubricant composition. Because the present work did not directly quantify reaction kinetics, graphite is discussed mainly as a complementary solid lubricant that can support friction reduction and thermal lubrication stability during high-temperature compaction.

However, direct application of the lubricant contents used in previous Fe–MoS_2_–graphite composites to Fe–5.0 wt.%Si SMC compacts requires caution [12,19,20]. In SMC compacts, contact between metal particles and densification directly affect the final density and mechanical uniformity; therefore, excessive lubricant addition can hinder interparticle contact and cause increased porosity or reduced densification [6,8,12,13]. Graphite also has low density and soft characteristics; therefore, an increase in graphite content may cause density reduction and degradation of local mechanical properties [17,20].

Therefore, evaluation of the MoS_2_/graphite mixing ratio should be approached as composition design that considers friction reduction, densification uniformity, and local mechanical uniformity during high-temperature compaction, rather than simply identifying the lowest friction coefficient [12,13,19,20]. The MoS_2_/graphite mixing conditions in this study were designed by referring to the combined lubrication effect reported in Fe–MoS_2_–graphite composites and the lubricant content range validated in the previous Fe–5.0 wt.%Si SMC study by Kang and Lee [12,19,20]. In that previous study, MoS_2_ was examined at 0.75, 1.0, and 1.25 wt.%, and graphite was examined at 0.3 and 0.5 wt.% [12]. Kang and Lee reported that low H_3_PO_4_ and PI contents combined with 0.3 wt.% graphite provided a favorable balance of density, permeability, and Q-value [12]. However, MoS_2_ and graphite were evaluated as separate lubricant conditions in that study, whereas the present study examines combined MoS_2_/graphite mixing ratios.

Based on this previous composition range, 0.75 wt.% MoS_2_ was selected as the central level in the present study. A lower level of 0.5 wt.% MoS_2_ was included to evaluate a reduced-lubricant condition, and 1.0 wt.% MoS_2_ was selected as the higher comparison level while avoiding excessive MoS_2_ addition. For graphite, 0.3 and 0.5 wt.% were selected based on the previous validated range, and 0.8 wt.% was additionally included to examine the effect of increased graphite content on densification and local mechanical uniformity. Accordingly, nine lubricant compositions were designed to evaluate the effects of the combined MoS_2_ and graphite mixing ratio on friction behavior, forming resistance, densification uniformity, and local mechanical uniformity. H_3_PO_4_ and PI were fixed at 0.25 wt.% each to maintain constant insulation and binder conditions [12,21,22,23].

The research procedure consisted of lubricant composition selection, toroidal-core-based finite element analysis (FEA), and microstructural analysis of the selected condition. First, rectangular specimens were fabricated using a 2-Press 1-Anneal (2P1A) process for the nine lubricant compositions by referring to the staged pressing and heat-treatment process proposed in previous Fe–Si-based SMC studies, and temperature-dependent friction coefficient tests and Vickers hardness tests were performed [6,7,12,13]. These tests were conducted to evaluate friction reduction behavior and local mechanical uniformity.

The friction coefficient test results were applied as FEA input conditions to simulate the forming behavior of a bulk-type toroidal core, and the compaction analysis was performed using the DEFORM-3D tool. The DEFORM-3D simulation used a half-core model of the toroidal core considering analysis efficiency and geometric symmetry. In the analysis, the relative density distribution, hydrostatic stress, and effective strain distribution were compared according to lubricant composition [11,24,25].

A toroidal core was fabricated using the selected lubricant composition obtained from the analysis, and XRD and SEM-EDS analyses were performed. XRD analysis was conducted to identify the formation of secondary phases other than the Fe–Si matrix after high-temperature compaction and annealing [12,25]. SEM-EDS analysis was conducted to examine the pore distribution, interparticle bonding state, and additive element distribution inside the toroidal specimen [12,25]. Based on these results, a MoS_2_/graphite lubricant composition suitable for high-temperature compaction of Fe–5.0 wt.%Si SMCs is proposed.

It was hypothesized that a balanced MoS_2_/graphite lubricant composition would provide a complementary lubrication effect during high-temperature compaction, based on previous studies on MoS_2_/graphite solid lubricants and Fe–MoS_2_-based composites [14,15,16,17,19,20]. MoS_2_ was expected to reduce interparticle and die-wall friction through its low interfacial shear resistance [14,15,16,20]. Graphite was expected to complement the lubricating role of MoS_2_ at high temperature [17,20] and delay the reaction between MoS_2_ and the Fe matrix [19,20]. Therefore, an appropriate MoS_2_/graphite mixing ratio was expected to reduce friction and forming resistance while improving densification uniformity and local mechanical uniformity.

Based on this hypothesis, the significance of this study lies in the composition-based evaluation of MoS_2_/graphite solid lubricants for Fe–5.0 wt.%Si SMC compaction under fixed insulation and binder conditions [12,21,22,23]. Previous studies have mainly examined individual lubricant effects in Fe–5.0 wt.%Si SMCs or general Fe–MoS_2_–graphite self-lubricating composites [12,19,20]. In contrast, this study systematically evaluates combined MoS_2_/graphite mixing ratios in terms of friction behavior, forming resistance, densification uniformity, local mechanical uniformity, and microstructural characteristics. This approach provides a practical basis for selecting lubricant compositions for high-temperature compaction of Fe–Si-based SMCs, where both friction reduction and uniform densification are required [6,8,12,13].

## 2. Materials and Methods

### 2.1. Materials and Specimen Preparation

#### Powder Materials and Lubricant Composition Design

In this study, a laboratory-developed Fe–5.0 wt.%Si alloy powder prepared in-house was used as the base powder. The powder composition was Fe 94.85 wt.%, Si 5.02 wt.%, and O 0.13 wt.%, as reported in the previous study by Kang and Lee [12]. The particle size distribution of the Fe–5.0 wt.%Si powder was measured using a particle size analyzer (Mastersizer 3000, Malvern Instruments Ltd., Malvern, UK). The Dv(10), Dv(50), and Dv(90) values were 38.058 μm, 76.247 μm, and 141.126 μm, respectively, and the volume-weighted mean diameter D [3,4] was 83.600 μm. To evaluate the effect of lubricant composition on the friction behavior and mechanical uniformity of Fe–5.0 wt.%Si SMCs, MoS_2_ and graphite were used as solid lubricants [14,16,17,19,20]. The MoS_2_ powder had a particle size of approximately 3 μm. The graphite powder had a particle size of less than 20 μm. H_3_PO_4_ and polyimide (PI) were fixed at 0.25 wt.% each to maintain constant insulation and binder conditions [12,21,22,23].

For the H_3_PO_4_ coating, H_3_PO_4_ corresponding to 0.25 wt.% of the total Fe–5.0 wt.%Si powder mass was stirred in acetone for approximately 3 min. The Fe–5.0 wt.%Si powder was then immersed in the H_3_PO_4_–acetone solution, maintained at 90 °C for 60 min, and cooled to room temperature. After cooling, the powder was washed three times with acetone to remove unreacted H_3_PO_4_ remaining in the powder. The H_3_PO_4_-coated Fe–5.0 wt.%Si powder was finally dried at 150 °C for 30 min.

After the H_3_PO_4_ coating, the H_3_PO_4_-coated Fe–5.0 wt.%Si powder was immersed in a dichloromethane solution containing 0.25 wt.% PI. The powder was then coated and dried using a dryer, followed by additional drying at 120 °C for 15 min. In this process, immersion in acetone and dichloromethane solutions was used to form relatively uniform phosphate and PI layers on the Fe–5.0 wt.%Si powder surface [12,22,23].

The MoS_2_ and graphite contents were determined by referring to the lubricant content range used in a previous Fe–5.0 wt.%Si SMC study [12]. MoS_2_ was set at 0.5, 0.75, and 1.0 wt.%, and graphite was set at 0.3, 0.5, and 0.8 wt.%. Accordingly, a total of nine MoS_2_/graphite lubricant compositions were prepared. After the H_3_PO_4_ and PI coating steps, MoS_2_ and graphite powders were added to the coat-ed Fe–5.0 wt.%Si powder according to the designed lubricant compositions. Because MoS_2_ and graphite are powder-type solid lubricants, the coated Fe–5.0 wt.%Si powder and solid lubricants were mechanically mixed. To reduce local agglomeration of the solid lubricants, agglomerated lubricant particles were crushed using a mortar and pestle before mixing. The powder mixture was then stirred using the mortar and pestle for 10 min and additionally stirred in a plastic mixing container for 5 min to improve homogenization of the lubricant components. Each composition is listed in Table 1.

### 2.2. Experimental Evaluation of Rectangular Specimens

#### 2.2.1. Friction Coefficient Measurement and Exponential Extrapolation

**Figure 1:**
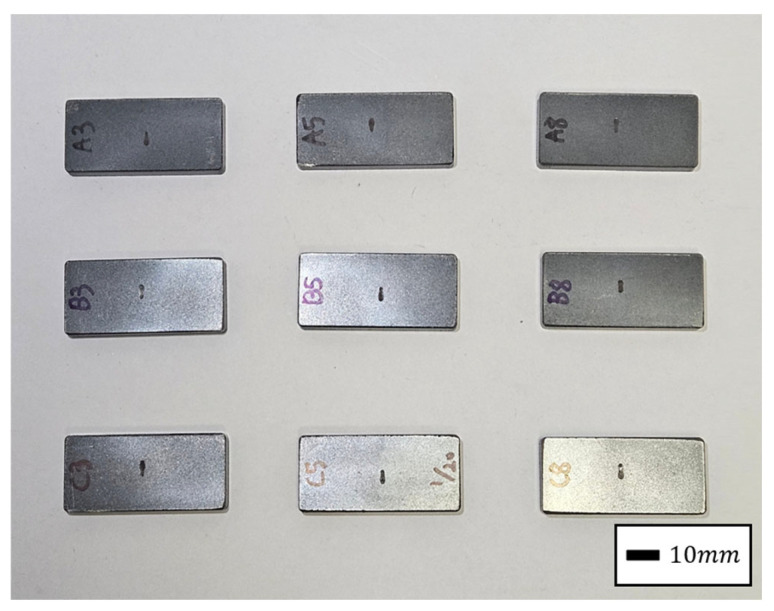
Rectangular Fe–5.0 wt.%Si SMC specimen after friction coefficient testing. Scale bar: 10 mm.

To evaluate the friction behavior according to lubricant composition, temperature dependent friction coefficient tests were conducted using a multifunctional tribometer (UMT-TRIBOLAB, Bruker, Ettlingen, Germany) according to ASTM G133 [14,19,26]. The test was performed using a reciprocating ball-on-plate method, in which a SUJ2 steel ball with a diameter of 9.525 mm, corresponding to a radius of 4.7625 mm, was used as the counter body, and the rectangular Fe–5.0 wt.%Si SMC specimen was used as the plate specimen. Before the test, the surface conditions of the lower and upper specimens were checked, and the lower specimen was fixed to the test stage to prevent movement during testing. The normal load was set to 21 N. The reciprocating frequency and stroke length were set to 1 Hz and 3 mm, respectively. Based on these conditions, the average sliding speed was calculated as 6 mm/s. The test duration was 90 min, and the total sliding distance was calculated as 32.4 m. The tests were conducted at room temperature (RT), 150 °C, and 350 °C. Before each test, the target temperature was maintained for at least 5–10 min to stabilize the test condition. The initial shape of the rectangular specimen used for the friction coefficient test is shown in Figure 1. The measured temperature-dependent friction coefficients were extrapolated using an exponential function to estimate the friction coefficient at 550 °C, which corresponds to the actual second high-temperature forming condition [16,17]. The temperature-dependent friction coefficient was expressed as follows:

(1) $$\mu \left(T\right)=ae^{bT}$$

where *μ(T)* is the friction coefficient at temperature T, and a and b are fitting constants. The extrapolated friction coefficient at 550 °C was used as the interface friction condition in the DEFORM-3D compaction simulation for relative comparison among lubricant compositions.

**Figure 2:**
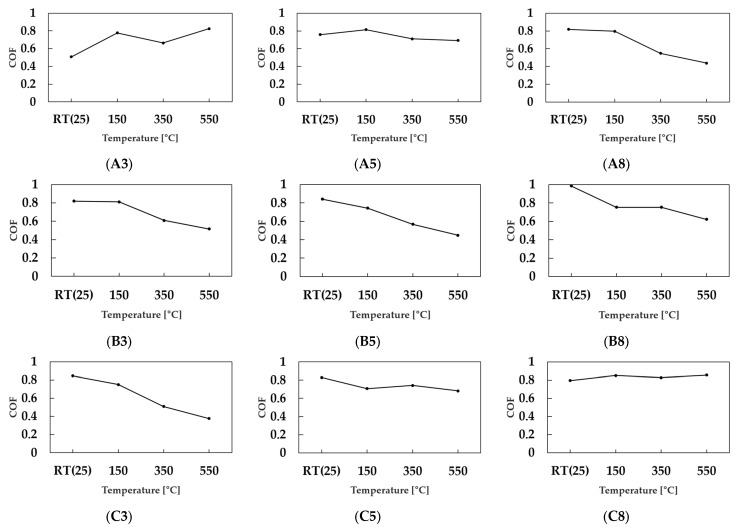
Temperature-dependent friction coefficients of Fe–5.0 wt.%Si SMC specimens with different MoS_2_/graphite lubricant compositions. A, B, and C denote MoS_2_ contents of 0.5, 0.75, and 1.0 wt.%, respectively, while 3, 5, and 8 denote graphite contents of 0.3, 0.5, and 0.8 wt.%, respectively.

Figure 2 shows the change in friction coefficient of the Fe–5.0 wt.%Si SMC specimens according to the MoS_2_/graphite lubricant composition. The friction coefficient results for each case are summarized in Table 2.

In this result, the friction coefficient did not decrease uniformly with increasing tem-perature for all compositions. Some compositions showed a decrease in friction coefficient as the temperature increased, whereas others showed an increase in specific temperature ranges. This indicates that the lubrication behavior under high-temperature contact condi-tions varied depending on the MoS_2_/graphite mixing ratio [14,16,17,19]. The C3 condition showed the lowest friction coefficient among the nine lubricant compositions. In particular, C3 exhibited a friction coefficient of 0.509 at 350 °C, indicating the lowest interfacial friction resistance under high-temperature contact conditions. In addition, the friction coefficient of C3 at 550 °C was calculated as 0.376875, showing favorable lubrication behavior under high-temperature forming conditions. This result indicates that the combination of 1.0 wt.% MoS_2_ and 0.3 wt.% graphite is an effective lubricant composition for reducing high-temperature friction in Fe–5.0 wt.%Si SMCs.

In particular, the C3 condition showed a decreasing trend in friction coefficient with increasing temperature, indicating reduced friction resistance at the powder–die interface under high-temperature forming conditions [16,17]. Based on these results, the first press-ing step at 350 °C was set as the pre-densification step, and the second pressing step at 550 °C was set as the final densification step for Toroidal core compaction.

#### 2.2.2. Vickers Hardness Measurement and Hardness Uniformity Evaluation

Vickers hardness tests were conducted to evaluate the local mechanical uniformity according to lubricant composition. The test was performed on the rectangular Fe–5.0 wt.%Si SMC specimens shown in Figure 1 after completion of the 2P1A process and annealing. Vickers hardness was measured using a Vickers hardness tester (FV-810, FU-TURE-TECH CORP., Kawasaki, Japan), and the test was conducted according to KS B 0811 [27]. The test load was set to 1 kgf, and the dwell time was set to 10 s.

**Figure 3:**
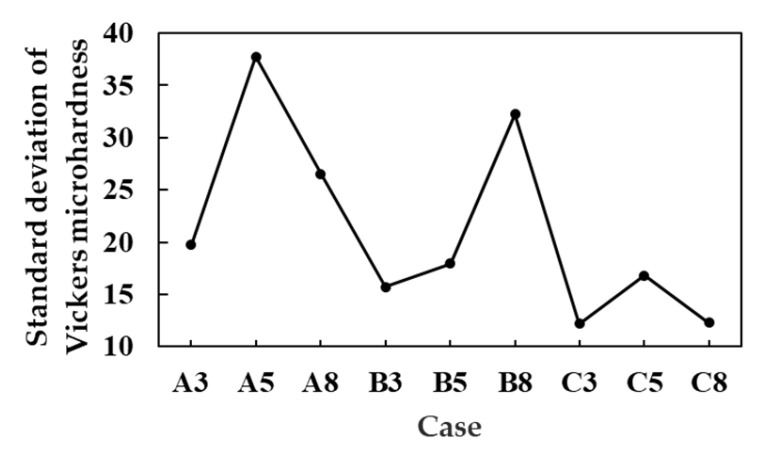
Standard deviation of Vickers hardness values measured at 10 points for each MoS_2_/graphite lubricant composition.

A total of 10 Vickers hardness points were measured for each specimen. The measurement positions were distributed at different locations on the specimen surface to ex-amine the positional hardness variation. The individual Vickers hardness values measured at 10 points for each lubricant composition are summarized in Table 3. These data were used to calculate the standard deviation values shown in Figure 3 and to compare the positional hardness variation among the different lubricant compositions. The two diagonal lengths of the indentation formed at each point were measured, and Vickers hardness was calculated using the average diagonal length according to the following equation:

(2) $$HV=\;1.8544\frac{F}{d^{2}}$$

where HV is the Vickers hardness, F is the test load (kgf), and d is the average diagonal length of the indentation (mm). In this study, F = 1 kgf was applied.

The standard deviation of the 10 point measurements was calculated for each case. In this study, the Vickers hardness standard deviation was used as the main comparison index. The Vickers hardness standard deviation represents the positional hardness scatter within the specimen, and a lower value was interpreted as indicating higher local mechanical uniformity. This evaluation was used to compare the effect of the MoS_2_/graphite lubricant composition on the local hardness uniformity of the Fe–5.0 wt.%Si SMC specimens.

To evaluate whether the Vickers hardness values differed significantly among the nine lubricant compositions, a one-way analysis of variance (ANOVA) was performed using the 10-point Vickers hardness data for each case. In the ANOVA, the lubricant composition was used as the grouping factor, and the Vickers hardness value was used as the response variable. The ANOVA was performed using the following equations:

(3) $$F=\;\frac{MS_{between}}{MS_{within}}$$

(4) $$MS_{between}=\frac{SS_{between}}{k-1}$$

(5) $$MS_{within}=\frac{SS_{within}}{N-k}$$

where $SS_{between}$ is the sum of squares due to the difference in the average hardness values among the nine lubricant compositions, and $SS_{within}$ is the sum of squares due to the scatter of the 10-point hardness values within each composition. $MS_{between}$ and $MS_{within}$ are the mean squares between groups and within groups, respectively. The F-value indicates whether the difference among the lubricant compositions is sufficiently large compared with the scatter within each composition. In this study, (k = 9) represents the number of lubricant composition groups, and (N = 90) represents the total number of Vickers hardness measurements. A significance level of (*p* < 0.05) was applied.

To evaluate whether the Vickers hardness values differed significantly among the nine lubricant compositions, a one-way ANOVA was performed, and the results are summarized in Table 4. The calculated F-value was 4.245, which was higher than the F critical value of 2.055, and the *p*-value was 0.000273. Since the *p*-value was lower than the significance level of 0.05, the Vickers hardness values were considered to be significantly different among the nine lubricant compositions. This result indicates that the MoS_2_/graphite lubricant composition had a statistically significant effect on the Vickers hardness response. However, in this study, the standard deviation of the 10-point Vickers hardness values was used as the main index for comparing local mechanical uniformity.

Figure 3 shows the standard deviation of the 10 points Vickers hardness measurements for each MoS_2_/graphite lubricant composition. In this study, the mechanical uniformity of the compact was compared with a focus on the hardness scatter according to measurement position. A lower Vickers hardness standard deviation indicates a smaller hardness difference depending on the measurement position and a more uniform mechanical response within the compact.

Comparison of the standard deviation for each case showed that the C3 condition had the lowest value. The Vickers hardness standard deviation of C3 was 12.17177, which was the lowest among the nine lubricant compositions. This indicates that the hardness variation according to measurement position was the smallest under the C3 condition and that C3 showed the best result in terms of Vickers hardness uniformity. The Vickers hardness standard deviation result was used as experimental evidence supporting the mechanical uniformity of the C3 condition.

### 2.3. Finite Element Analysis

#### 2.3.1. Toroidal Compaction Simulation Model

DEFORM-3D compaction simulation was performed to evaluate the effect of friction coefficient variation according to lubricant composition on the forming behavior of the Toroidal core [11,24,25]. The analysis target was a Toroidal specimen with an outer diameter of 20.38 mm, an inner diameter of 12.60 mm, and a final height of 6.72 mm. The simulation model was constructed based on the previous Toroidal core compaction analysis conditions, and a half-core model was used considering analysis efficiency and geometric symmetry [24]. The temperature-dependent friction coefficient results measured from the rectangular specimens were applied as friction conditions at the forming interfaces for each lubricant composition [26].

First, a compaction simulation was performed under the 350 °C forming condition until the Toroidal specimen reached the final height of 6.72 mm, and the final reaction load generated at this point was defined as the reference load. Subsequently, 50% of the reference load was applied as the first pressing condition, and the second pressing step was performed at 550 °C until the final height of 6.72 mm was reached. This simulation condition was applied equally to all lubricant composition cases.

#### 2.3.2. Evaluation Criteria for Simulation Results

The FEA results were compared based on the relative density Max–min value, effective strain Max–min value, and hydrostatic stress Max–min value [11,24,25]. The relative density Max–min value was used as an index to evaluate the positional density difference inside the Toroidal core. A smaller value was interpreted as indicating higher densification uniformity within the compact.

The effective strain Max–min value was used as an index to examine local strain concentration and powder rearrangement behavior during compaction [24,25]. A larger effective strain Max–min value was interpreted as indicating strain concentration in a specific region.

The hydrostatic stress Max–min value was used as an index to examine the compressive stress state and densification driving-force transfer inside the compact [11,24,25]. A smaller hydrostatic stress Max–min value was interpreted as indicating a more stable compressive stress distribution and relatively uniform transfer of the densification driving force within the compact.

Finally, the forming behavior according to lubricant composition was evaluated by comparing the relative density Max–min value, effective strain Max–min value, and hy-drostatic stress Max–min value together. Through this evaluation, a lubricant composition with favorable characteristics in terms of densification uniformity, local strain concentration, and compressive stress distribution stability was selected.

### 2.4. Characterization of Selected Lubricant Condition

The selected lubricant composition was determined by considering the Vickers hard-ness test and FEA results together. XRD analysis using a SmartLab X-ray diffractometer (Rigaku Corporation, Tokyo, Japan) and scanning electron microscopy-energy dispersive spectroscopy (SEM-EDS; JEOL Ltd., Tokyo, Japan) analysis were performed on the specimen fabricated with the selected lubricant composition [12,25].

XRD analysis was performed to examine whether secondary phases, such as residual lubricant phases, oxides, sulfides, or carbides, were formed at a detectable level in addition to the Fe–Si matrix after high-temperature compaction and annealing [12,19,28]. Through this analysis, the phase stability after the high-temperature process was evaluated for the selected lubricant composition.

SEM analysis was performed to examine the pore distribution, interparticle bonding state, and local densification state inside the specimen [12,25,28]. EDS analysis was mainly used to examine the local distribution of Mo, S, and C and to evaluate the distribution state of the MoS_2_/graphite lubricant components [12,14,19,28].

In this study, XRD and SEM-EDS analyses were performed as subsequent analyses to verify the phase stability and microstructural validity of the condition selected through the DEFORM-3D simulation [12,25].

## 3. Simulation Results

### 3.1. FEA Model

**Figure 4:**
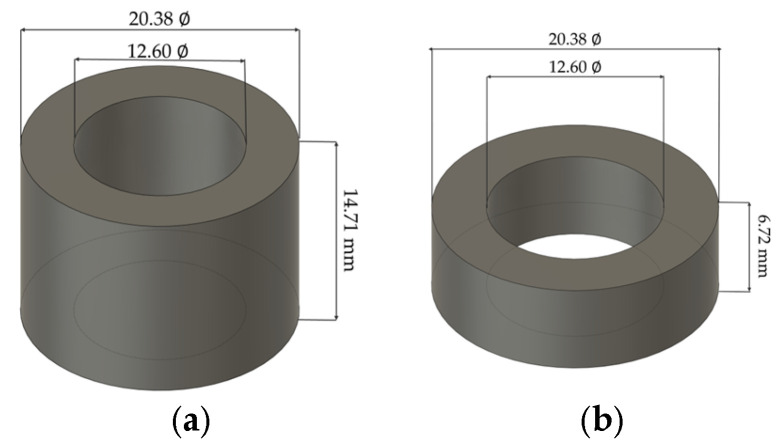
Finite element analysis (FEA) model for toroidal core compaction: (**a**) workpiece before compaction, (**b**) workpiece after compaction.

A finite element analysis (FEA) model was constructed using DEFORM-3D to simulate the forming behavior of the Fe–5.0 wt.%Si SMC Toroidal core [11,24,25]. Figure 4 shows the Toroidal workpiece model used in this study. In Figure 4, the Toroidal work-piece was modeled with an outer diameter of 20.38 mm, an inner diameter of 12.60 mm, and a final forming height of 6.72 mm. The initial powder filling height before compaction was set to 14.71 mm. The workpiece mesh of the FEA model consisted of 272,483 elements. The tap density of the Fe–5.0 wt.%Si powder was experimentally measured as 3.34 g/cm^3^, and the true density was 7.80 g/cm^3^. The initial relative density was calculated as 0.428 by dividing the tap density by the true density. The initial relative density was calculated using the following equation:

(6) $$\rho_{r}=\frac{\rho_{tap}}{\rho_{true}}$$

For each MoS_2_/graphite lubricant composition, the friction coefficient was applied as the contact friction condition between the workpiece and forming tools. In this model, the same composition-dependent friction condition was assigned to the workpiece–die, workpiece–upper punch, and workpiece–lower punch interfaces. The friction coefficient was used as a composition-dependent input value to compare the effect of lubricant composition on high-temperature forming behavior.

The FEA results were evaluated based on relative density, hydrostatic stress, and effective strain. Relative density was used as the main index for evaluating densification behavior and density uniformity of the compact. Hydrostatic stress was used as an index to examine the compressive stress state and densification driving force inside the compact. Effective strain was used as an auxiliary index to examine local strain concentration and powder rearrangement behavior during compaction.

### 3.2. Comparison of FEA Results

After completion of the FEA calculation, the numerical data were extracted from the final forming step using the DEFORM-3D post-processor. Only the workpiece region was selected for data extraction, excluding the die and punch components. For each lubricant composition, the distributions of relative density, effective strain, and hydrostatic stress were obtained at the final compacted state. The maximum and minimum values of each variable were then extracted from the post-processed numerical data, and the difference between these values was calculated as the Max–Min value. In this study, the Max–Min value was used as a quantitative index to compare the distribution range of each FEA var-iable among the different lubricant compositions.

**Figure 5:**
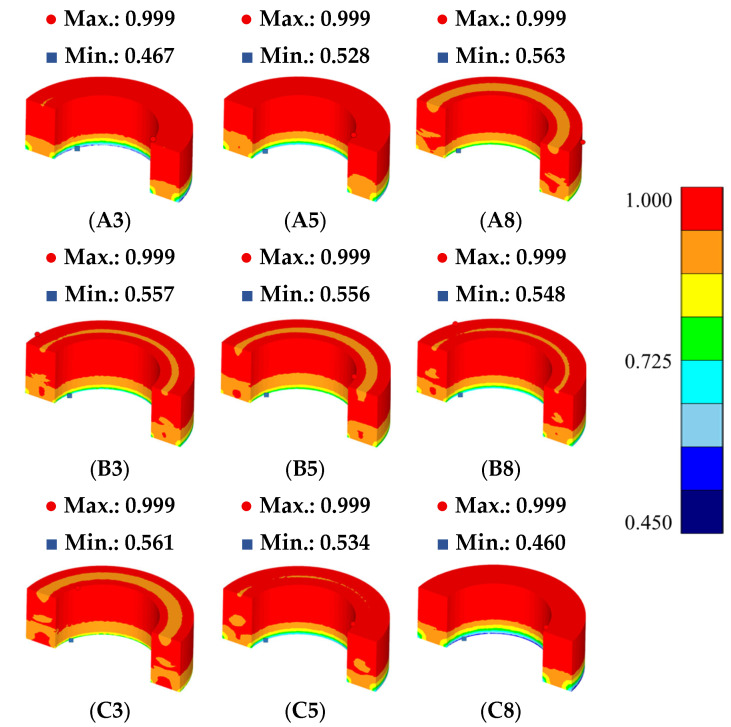
Relative density distributions of Fe–5.0 wt.%Si SMC Toroidal cores with different MoS_2_/graphite lubricant compositions obtained from DEFORM-3D. A, B, and C denote MoS_2_ contents of 0.5, 0.75, and 1.0 wt.%, respectively, while 3, 5, and 8 denote graphite contents of 0.3, 0.5, and 0.8 wt.%, respectively.

Figure 5 shows the relative density distribution of the Fe–5.0 wt.%Si SMC Toroidal compact obtained from the FEA results [11,24,25]. In this model, the lower punch was fixed, and only the upper punch moved downward to compress the workpiece. Under this uniaxial compaction condition, the relative density inside the compact can show different distributions along the height and radial directions [24,29]. Therefore, the relative density distribution was compared for each lubricant composition to evaluate the densification uniformity of the Toroidal compact.

In Figure 5, the relative density distribution of each case shows that the densification behavior inside the compact varied depending on the lubricant composition. Relative den-sity is a main index representing the local densification state inside the compact [11,24,25,29]. In this study, the relative density Max–min value was used to quantitatively compare the relative density distribution of each case. The relative density Max–min value was defined as the difference between the maximum and minimum relative density values inside the compact.

Among the analysis conditions, A8 showed the lowest relative density Max–min value of 0.437. C3 showed a relative density Max–min value of 0.439, and the difference from A8 was 0.002. Therefore, C3 was considered to exhibit a level of densification uni-formity similar to that of A8 based on the relative density Max–min value. This result shows that the C3 condition exhibited stable densification behavior in terms of the relative density distribution of the Toroidal compact.

**Figure 6:**
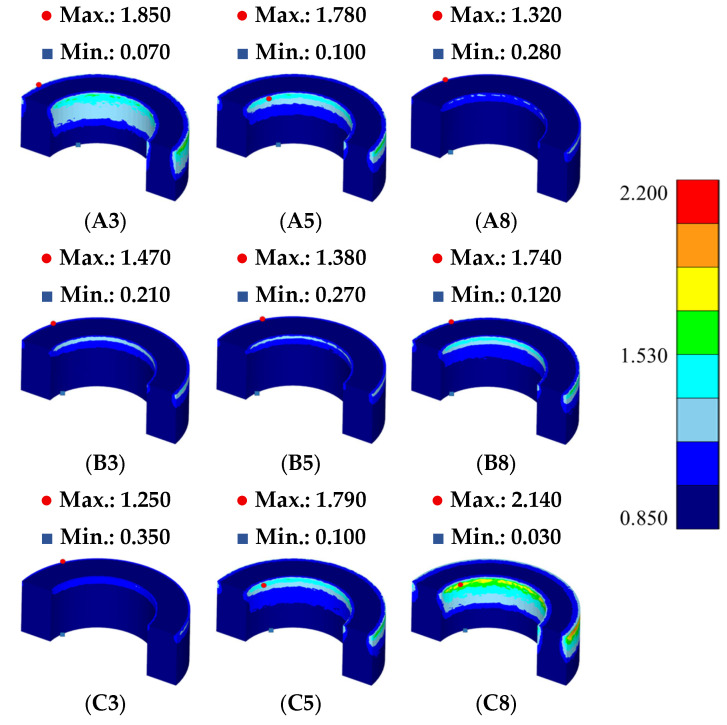
Effective strain distributions of Fe–5.0 wt.%Si SMC Toroidal cores with different MoS_2_/graphite lubricant compositions obtained from DEFORM-3D. A, B, and C denote MoS_2_ contents of 0.5, 0.75, and 1.0 wt.%, respectively, while 3, 5, and 8 denote graphite contents of 0.3, 0.5, and 0.8 wt.%, respectively.

Figure 6 shows the effective strain distribution of the Fe–5.0 wt.%Si SMC Toroidal compact after compaction [11,24,25]. Effective strain was used as an index to examine the local deformation behavior generated inside the workpiece during compaction. In powder compaction, effective strain can be interpreted as the accumulated equivalent strain associated with particle rearrangement, local deformation of powder particles, and pore closure during densification. Therefore, effective strain does not directly represent porosity itself, but it provides a deformation-based indication of regions where deformation related to porosity reduction is concentrated. In Figure 6, the effective strain was not uniformly distributed throughout the compact, and relatively high values appeared near the upper punch contact region and the inner and outer corner regions of the Toroidal geometry.

This effective strain distribution is associated with the upper punch loading condi-tion, restricted particle flow caused by the Toroidal geometry, and friction conditions at the workpiece–die interface [24,29]. In particular, the inner and outer corner regions are locations where particle movement is restricted during compaction; therefore, local defor-mation can be relatively high. Regions with relatively high effective strain can be inter-preted as locations where particle rearrangement and pore closure proceeded intensively owing to restricted powder flow and local deformation. Conversely, large spatial differ-ences in effective strain indicate nonuniform deformation behavior, which can be associ-ated with nonuniform pore closure during compaction. Therefore, the effective strain dis-tribution was compared for each lubricant composition to evaluate the degree of local strain concentration inside the compact.

To compare the uniformity of the effective strain distribution, the difference between the maximum and minimum effective strain values was defined as the effective strain Max–min value [24,25]. A smaller effective strain Max–min value was interpreted as indi-cating a smaller positional strain difference inside the compact and lower local strain concentration in specific regions. In terms of powder porosity, a lower effective strain Max–min value indicates that the deformation related to powder rearrangement and pore closure was more uniformly distributed within the compact. Among the analysis conditions, C3 showed the lowest effective strain Max–min value of 0.900. This means that C3 exhibited the lowest effective strain variation among the nine lubricant compositions and showed the lowest local strain concentration during compaction. This result suggests that the C3 condition provided a more uniform deformation state related to pore closure and densification during Toroidal core compaction.

**Figure 7:**
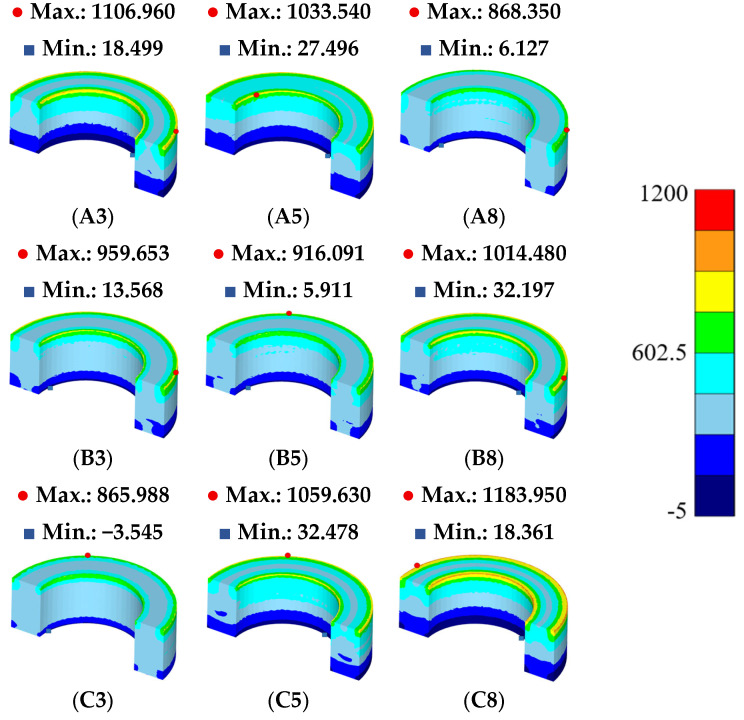
Absolute hydrostatic stress distributions of Fe–5.0 wt.%Si SMC Toroidal cores with different MoS_2_/graphite lubricant compositions obtained from DEFORM-3D. A, B, and C denote MoS_2_ contents of 0.5, 0.75, and 1.0 wt.%, respectively, while 3, 5, and 8 denote graphite contents of 0.3, 0.5, and 0.8 wt.%, respectively.

Figure 7 shows the hydrostatic stress distribution of the Fe–5.0 wt.%Si SMC Toroidal core according to the MoS_2_/graphite lubricant composition [11,24,25,29,30]. Hydrostatic stress was used as an index to examine the compressive stress state and densification driving force acting inside the compact during powder compaction [24,25,29,30]. For easier compari-son among the lubricant compositions, the hydrostatic stress distribution in Figure 7 was displayed based on the absolute magnitude of the stress. However, because the hydrostatic stress generated during compaction corresponds to a compressive stress state, the numer-ical hydrostatic stress values that had been expressed as positive values in the original manuscript were denoted with negative signs in the revised manuscript to preserve the physical meaning of compressive hydrostatic stress. In this study, the hydrostatic stress Max–min value of each case was compared to evaluate the uniformity of the positional compressive stress distribution [24,25,29,30].

The hydrostatic stress Max–min value was the lowest in the A8 case, with a value of 862.223 MPa. This indicates that the positional variation in hydrostatic stress inside the Toroidal core was the smallest under the A8 condition. The hydrostatic stress Max–min value of the C3 case was 869.533 MPa, which was the second-lowest value among the nine lubricant compositions. The difference in hydrostatic stress variation between A8 and C3 was 7.310 MPa, and both conditions showed relatively uniform compressive stress distributions.

A condition with a smaller hydrostatic stress variation can be interpreted as a condi-tion in which compressive stress was not excessively concentrated in a specific region during compaction and the densification driving force was transferred relatively uniform-ly [24,25,29,30]. The A8 case showed the best result in terms of hydrostatic stress distribu-tion uniformity, and the C3 case also exhibited a similar level of hydrostatic stress distri-bution stability.

In particular, the C3 case showed the lowest friction coefficient and the lowest stand-ard deviation in the Vickers hardness test, indicating the highest positional hardness uni-formity. In addition, C3 showed a low variation in the effective strain distribution. There-fore, the hydrostatic stress result provides additional support for the mechanical forming stability of the C3 condition.

## 4. Characterization of the Selected Lubricant Composition

### 4.1. XRD Analysis

**Figure 8:**
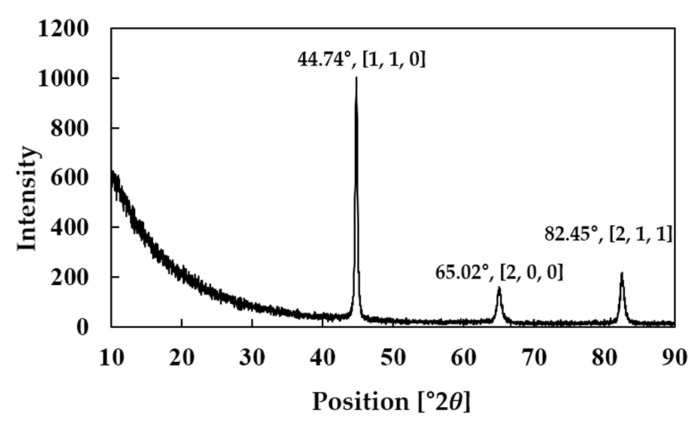
XRD pattern of the Fe–5.0 wt.%Si SMC Toroidal core fabricated using the C3 lubricant composition with 1.0 wt.% MoS_2_ and 0.3 wt.% graphite.

Figure 8 shows the XRD pattern of the Fe–5.0 wt.%Si SMC Toroidal core fabricated using the C3 lubricant composition, which was selected as the final lubricant condition [12,25,31]. The C3 condition corresponds to the lubricant composition containing 1.0 wt.% MoS_2_ and 0.3 wt.% graphite. XRD analysis was performed using a SmartLab X-ray dif-fractometer (Rigaku Corporation, Tokyo, Japan). The XRD measurement was conducted on the surface of the bulk C3 specimen after compaction and annealing to examine whether a phase change occurred in the Fe-based matrix or whether additional crystalline phases were formed during the high-temperature process.

The XRD results showed major diffraction peaks near 2θ = 44.74°, 65.02°, and 82.45°. These peaks correspond to the (1, 1, 0), (2, 0, 0), and (2, 1, 1) planes of the α-Fe-based bcc Fe–Si matrix, respectively [31,32]. Among these peaks, the (1, 1, 0) peak near 44.74° showed the highest intensity, indicating that the main crystalline phase of the Fe–5.0 wt.%Si SMC To-roidal core fabricated using the C3 condition was the α-Fe-based bcc matrix [31,32].

In the Fe–5.0 wt.%Si composition, Si exists as a solid solution in the Fe matrix, and α-Fe-based bcc diffraction peaks were dominant in the XRD pattern [31,32]. This result in-dicates that the bcc crystal structure of the Fe–Si matrix was maintained after high-temperature compaction and annealing under the C3 condition with 1.0 wt.% MoS_2_ and 0.3 wt.% graphite [12,25,31]. In addition, no distinct additional crystalline peaks that could be clearly assigned to FeS or oxide phases were observed. Because conventional XRD provides averaged phase information from the analyzed surface area, local phases associated with small reaction products or residual lubricant-related regions could not be directly identified by this measurement. Therefore, local regions were not assigned to specific phases in this study. Accordingly, the phase assignment in this study was limited to the α-Fe-based bcc Fe–Si matrix observed in the XRD results.

### 4.2. SEM-EDS Analysis

**Figure 9:**
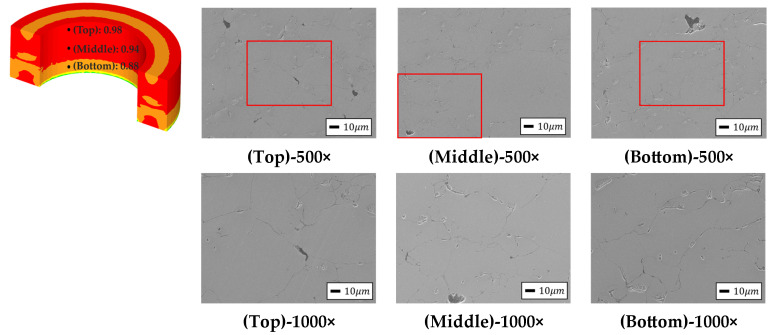
FEA-based relative density values and corresponding SEM micrographs of the Fe–5.0 wt.%Si SMC Toroidal core at the Top, Middle, and Bottom regions, observed at 500× and 1000× magnifications. In the FEA contour image, the different colors indicate the relative density distribution, with representative values of 0.98, 0.94, and 0.88 at the Top, Middle, and Bottom regions, respectively. The red-marked regions in the 500× images indicate the areas observed at 1000× magnification.

Figure 9 shows the FEA-based relative density values at the Top, Middle, and Bottom regions of the Toroidal core fabricated using the selected C3 condition and the corre-sponding SEM images [12,25,28,32,33]. To provide a clearer microstructural comparison, SEM micrographs were presented at both 500× and 1000× magnifications. The 500× imag-es were used to observe the overall densification state in each region, whereas the 1000× images were added to examine the particle boundaries and interparticle contact regions in more detail. In the 500× SEM images, the regions marked in red were further observed at 1000× magnification to examine the particle-boundary morphology more clearly. In the FEA results, the relative density values were 0.98 at the Top region, 0.94 at the Middle re-gion, and 0.88 at the Bottom region. In addition, the average FEA-based relative density of the C3 compact was 0.936. SEM observations were performed using scanning electron microscopy (SEM; JEOL Ltd., Tokyo, Japan).

The SEM observations showed densely compacted Fe–Si powder particles in the Top, Middle, and Bottom regions [12,25,28,32,33]. In each region, interparticle contact regions were formed relatively continuously, and a stable densification state was observed overall [12,25,32,33]. The higher-magnification SEM images further confirmed that the particle boundaries were distinguishable and that the powder particles were in close contact after compaction. In particular, although the Bottom region showed the lowest FEA-based rela-tive density among the three positions, the SEM image confirmed that the powder particles maintained a sufficiently compacted microstructure.

Therefore, the results in Figure 9 indicate that the SEM observations and FEA results for the Top, Middle, and Bottom regions of the Toroidal core consistently confirmed the densified microstructure under the C3 lubricant composition [12,25,32,33]. The additional 1000× SEM images provide more detailed evidence of the particle-boundary morphology and interparticle contact state, supporting the microstructural interpretation of the com-pacted C3 specimen.

**Figure 10:**
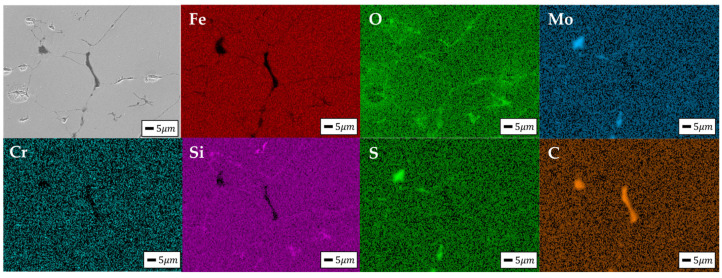
SEM-EDS elemental mapping images of the Fe–5.0 wt.%Si SMC Toroidal core fabricated using the selected lubricant composition: SEM image, Fe, O, Mo, Cr, Si, S, and C elemental maps.

Figure 10 shows the SEM-EDS elemental mapping results of the Fe–5.0 wt.%Si SMC Toroidal core fabricated using the selected C3 condition [12,25,28,34,35].

The EDS mapping results showed that Fe and Si were distributed throughout the analysis region, indicating that the Fe–Si matrix formed the main matrix phase of the specimen [12,28,31,32]. O showed relatively strong signals in some regions, confirming the presence of oxygen-containing regions inside the specimen after high-temperature com-paction and annealing [21,22,23,28,34].

Mo and S signals were observed in some local regions rather than being strongly and uniformly distributed throughout the entire region [19,28,35]. This indicates that the MoS_2_-based lubricant component included in the C3 condition remained in small amounts or locally inside the specimen after the high-temperature process [19,28,35]. In particular, the regions where Mo and S signals were observed together with O signals suggest that the MoS_2_-based lubricant component may be associated with oxygen-containing reaction regions during the high-temperature process [19,28,35]. Although Mo and S signals were locally observed in the SEM-EDS mapping results, these signals were interpreted as Mo/S-rich residual regions rather than direct evidence of a specific sulfide phase, because SEM-EDS provides elemental distribution information but cannot identify crystalline phases by itself. This interpretation is consistent with the XRD results, in which no distinct additional crystalline peaks corresponding to FeS or oxide phases were observed.

In the C mapping result, carbon signals related to the graphite lubricant were ob-served, and relatively high C distribution appeared in some regions [14,17,20]. This can be interpreted as the retention of graphite components included in the C3 condition inside the specimen after high-temperature compaction [17,20].

Therefore, the SEM-EDS results in Figure 10 show that the Fe–Si matrix formed the main matrix phase under the C3 lubricant composition, and lubricant-related elements such as Mo, S, and C were locally distributed [12,19,28,35]. In addition, the local distribu-tions of O and Mo/S signals indicate that lubricant-related components may exist in asso-ciation with some oxygen-containing reaction regions during high-temperature compac-tion and annealing [19,28,35].

## 5. Conclusions

This study evaluated the effect of MoS_2_/graphite lubricant composition on the high-temperature forming behavior and microstructural characteristics of Fe–5.0 wt.%Si SMCs. A total of nine lubricant compositions were compared using friction coefficient tests, Vickers hardness measurements, finite element analysis (FEA), and microstructural analysis. The initial expectation of this study was that an appropriate MoS_2_/graphite lubricant composition would reduce friction and forming resistance while improving densification uniformity and local mechanical uniformity during high-temperature compaction. The obtained results generally confirmed this expectation, and no entirely opposite result was observed. Based on the combined evaluation, the C3 condition containing 1.0 wt.% MoS_2_ and 0.3 wt.% graphite was selected as the optimum lubricant composition within the in-vestigated composition range for Toroidal core compaction.

The C3 condition showed the lowest friction coefficient among the nine lubricant compositions, indicating effective friction reduction under high-temperature contact con-ditions. In addition, C3 showed the lowest standard deviation in the Vickers hardness test, confirming the highest positional hardness uniformity. Compared with the highest values among the nine lubricant compositions, the C3 condition reduced the friction coefficient by 38.5% at 350 °C and by 55.9% at the extrapolated forming temperature of 550 °C. The Vick-ers hardness standard deviation was also reduced by 67.8%, indicating improved local mechanical uniformity. These experimental results indicate that the C3 condition is favorable in terms of friction reduction behavior and local mechanical uniformity.

The FEA results also supported the forming stability of the C3 condition. In the rela-tive density distribution, A8 showed a Max–min value of 0.437, while C3 showed a Max–min value of 0.439, with a difference of only 0.002. In the hydrostatic stress distribution, A8 showed a Max–min value of 862.223 MPa, while C3 showed a Max–min value of 869.533 MPa, with a difference of 7.310 MPa. These results indicate that A8 showed slightly lower variations in the relative density and hydrostatic stress distributions, but the differences from C3 were limited. In contrast, the effective strain Max–min value was the lowest under the C3 condition, indicating reduced local strain concentration and rela-tively uniform powder rearrangement during compaction. Compared with the highest values among the nine lubricant compositions, the relative density Max–min value, effec-tive strain Max–min value, and hydrostatic stress Max–min value of C3 were reduced by 18.6%, 57.3%, and 25.4%, respectively. In addition, the FEA-estimated total porosity of C3, calculated from the FEA-based average relative density of 0.936, was 6.4%. Therefore, C3 was selected by comprehensively considering the friction coefficient, Vickers hardness standard deviation, effective strain distribution, relative density distribution, and hydro-static stress distribution results.

XRD analysis of the Toroidal core fabricated using the C3 condition confirmed the main peaks of the α-Fe-based bcc Fe–Si matrix, indicating that the Fe–Si matrix structure was maintained after high-temperature compaction and annealing. SEM observations showed densely compacted Fe–Si powder particles in the Top, Middle, and Bottom regions, and interparticle contact regions were formed relatively continuously. SEM-EDS mapping showed locally distributed Mo, S, and C signals, indicating that MoS_2_- and graphite related lubricant components remained in some regions after the high-temperature process.

Overall, the C3 condition with 1.0 wt.% MoS_2_ and 0.3 wt.% graphite was determined to be the optimum lubricant composition for high-temperature compaction of Fe–5.0 wt.%Si SMCs within the investigated composition range. This condition provided a bal-anced combination of friction reduction behavior, positional hardness uniformity, forming stability, and microstructural compactness.

Future studies should further evaluate electromagnetic properties, including dynamic magnetic losses, core loss, and magnetic permeability, to clarify the functional performance of Fe–5.0 wt.%Si SMCs fabricated using the selected lubricant composition. In addition, advanced tribological tool wear models should be incorporated to consider the effects of contact pressure, sliding distance, frictional heating, and tool wear on interparticle friction and powder–tool interaction during repeated compaction.

## Figures and Tables

**Table 1:** Lubricant composition matrix of Fe–5.0 wt.%Si SMC specimens.

Case	MoS_2_ (wt.%)	Graphite (wt.%)	Total Lubricant Content (wt.%)
**A3**	0.5	0.3	0.8
**A5**	0.5	0.5	1.0
**A8**	0.5	0.8	1.3
**B3**	0.75	0.3	1.05
**B5**	0.75	0.5	1.25
**B8**	0.75	0.8	1.55
**C3**	1.0	0.3	1.3
**C5**	1.0	0.5	1.5
**C8**	1.0	0.8	1.8

**Table 2:** Measured and extrapolated friction coefficients for each lubricant composition.

Case	RT	150 °C	350 °C	Extrapolated 550 °C
**A3**	0.507	0.777	0.664	0.825221
**A5**	0.758	0.815	0.71	0.692652
**A8**	0.818	0.796	0.548	0.436991
**B3**	0.819	0.811	0.609	0.51597
**B5**	0.841	0.743	0.568	0.448048
**B8**	0.985	0.753	0.753	0.62174
**C3**	0.846	0.751	0.509	0.376875
**C5**	0.829	0.708	0.743	0.681924
**C8**	0.795	0.85	0.827	0.854322

**Table 3:** Vickers hardness data measured at 10 points for each lubricant composition.

Case	Point 1	Point 2	Point 3	Point 4	Point 5	Point 6	Point 7	Point 8	Point 9	Point 10	Standard Deviation
**A3**	150.9	169.2	162.1	192.1	219.1	145.8	170.1	168.2	174.3	168.7	19.7
**A5**	236	285.5	188.1	195.3	158	146.4	194.5	217	227.7	199.8	37.8
**A8**	203.6	234.7	173	168	148.8	182.7	142.2	160.6	155.7	159.1	26.5
**B3**	184.2	199.1	171.3	175.7	165.5	137.3	161.1	164.4	155.8	171.1	15.7
**B5**	148.2	137.6	167.1	149.3	134.8	150.1	151.1	146.4	196.9	131.3	17.9
**B8**	247.9	209.2	166.9	165.5	187.9	172.1	142.1	151.3	151.5	137.5	32.2
**C3**	157.7	174	153	182.9	170.1	172.6	182.2	157.5	156.8	145.5	12.1
**C5**	209	205.1	177.4	202.1	174.8	177.7	169.8	214.4	167.3	196.1	16.8
**C8**	157.8	160.8	150.5	153.4	182	163.5	168.7	136.6	176.2	161.8	12.3

**Table 4:** One-way ANOVA summary for the Vickers hardness data of the nine MoS_2_/graphite lubricant compositions.

Source of Variation	Sum of Squares (SS)	Degree of Freedom (df)	Mean Square (MS)	F-Value	*p*-Value	F Critical
Between groups	19,746.81	8	2468.351	4.245	0.000273	2.055
Within groups	47,095.78	81	581.429	-	-	-
Total	66,842.59	89	-	-	-	-

## Data Availability

The original contributions presented in the study are included in the article; further inquiries can be directed to the corresponding author.
